# Transcriptome analysis of *Lantana camara* flower petals reveals candidate anthocyanin biosynthesis genes mediating red flower color development

**DOI:** 10.1093/g3journal/jkad259

**Published:** 2023-11-17

**Authors:** Stephen Brooks Parrish, Dev Paudel, Zhanao Deng

**Affiliations:** Department of Environmental Horticulture, Gulf Coast Research and Education Center, University of Florida, IFAS, 14625 County Road 672, Wimauma, FL 33598, USA; Department of Environmental Horticulture, Gulf Coast Research and Education Center, University of Florida, IFAS, 14625 County Road 672, Wimauma, FL 33598, USA; Department of Environmental Horticulture, Gulf Coast Research and Education Center, University of Florida, IFAS, 14625 County Road 672, Wimauma, FL 33598, USA

**Keywords:** *Lantana camara*, transcriptome, anthocyanins, carotenoids, transcription factors, RNA-seq, flower color, Plant Genetics and Genomics

## Abstract

Flower color plays a crucial role in the appeal and selection of ornamental plants, directly influencing breeding strategies and the broader horticulture industry. *Lantana camara*, a widely favored flowering shrub, presents a rich palette of flower colors. Yet, the intricate molecular mechanisms governing this color variation in the species have remained largely unidentified. With the aim of filling this gap, this study embarked on a comprehensive de novo transcriptome assembly and differential gene expression analysis across 3 distinct lantana accessions, each showcasing a unique flower color. By harnessing the capabilities of both PacBio and Illumina sequencing platforms, a robust transcriptome assembly, encompassing 123,492 gene clusters and boasting 94.2% BUSCO completeness, was developed. The differential expression analysis unveiled 72,862 unique gene clusters that exhibited varied expression across different flower stages. A pronounced upregulation of 8 candidate core anthocyanin biosynthesis genes in the red-flowered accession was uncovered. This was further complemented by an upregulation of candidate *MYB75* (*PAP1*) and *bHLH42* (*TT8*) transcription factors. A candidate carotenoid cleavage dioxygenase (*CCD4a*) gene cluster also manifested a marked upregulation in white flowers. The study unveils the molecular groundwork of lantana's flower color variation, offering insights for future research and potential applications in breeding ornamental plants with desired color traits.

## Introduction

Flower color is a crucial component of both plant and human ecosystems, serving several important functions. Firstly, it acts as a beacon to attract pollinators such as bees, butterflies, and hummingbirds, which feed on the nectar and pollen, thereby aiding in the plant's reproductive process. Changes in flower color within a species can also impact the preferences of pollinators (Trunschke et al. 2021). Additionally, flowers play a significant role in human lives, providing vibrant landscapes that can improve moods and emotions (Haviland-Jones et al. 2005). As such, it is important for plant breeders to have a deep understanding of how flower color is produced within a particular species in order to cultivate flowers that will appeal to consumers.

Lantana (*Lantana camara*) is a popular flowering shrub in many countries, valued for its ornamental qualities, including prolific flowering and its ability to thrive in various landscapes, even under drought, salinity, diseases, and pest pressures. However, this species has become an invasive plant in some parts of the world, including Florida, where it is listed as a category 1 invasive species by the Florida Invasive Species Council (2019). To mitigate this problem, researchers at the University of Florida have developed 3 sterile lantana cultivars (Deng et al. 2017, 2020), while Ball FloraPlant has also introduced 3 sterile lantana varieties as part of their Bloomify series (Ball FloraPlant 2023a). These sterile cultivars are crucial to help prevent the spread of invasive lantana and protect the native lantana species (*Lantana depressa*) in Florida.

Lantanas are available in a variety of striking colors, including white, yellow, orange, red, pink, and rose. Among the various flower colors, red is the most popular, as shown by the numerous red cultivars offered by major breeding companies (Ball FloraPlant 2023b; Proven Winners 2023a; Syngenta Flowers 2023). The sterile cultivars also come in a range of colors, including red, orange, pink, mango, and rose (Ball FloraPlant 2023a; Proven Winners 2023b). However, to date, no sterile lantanas with white flowers have been introduced to the market.

The process of flower color development in plants is full of highly intricate biochemical reactions that have been extensively researched in various species (Tanaka et al. 2008; Cui et al. 2021; Wang et al. 2021). The dominant pigments responsible for flower coloration are carotenoids and anthocyanins (Berman et al. 2016; Wan et al. 2019). Carotenoids, found in a range of plant tissues, including flowers, fruits, and leaves, are responsible for producing hues of yellow, orange, and red (Zhao and Tao 2015). Anthocyanins, on the other hand, offer a more diverse range of colors, spanning from pale yellows to deep blues and purples (Liu et al. 2021). The combination of these 2 pigment classes contributes to the vibrant colors of flowering plants, with their presence varying according to the specific plant species.

The structural genes involved in carotenoid biosynthesis have been successfully identified in various plant species, including petunias, lilies, and peonies (Kishimoto et al. 2018; Wang and Yamagishi 2019; Zou et al. 2023). The biosynthesis of the red pigment lycopene from geranylgeranyl diphosphate requires several enzymes such as phytoene synthase (PSY), phytoene desaturase (PDS), ζ-carotene isomerase (Z-ISO), ζ-carotene desaturase (ZDS), and carotenoid isomerase (CRISTO). Following this, the pathway diverges and lycopene ε-cyclase (LCYE), lycopene β-cyclase (LCYB), cytochrome P450 family 97 A (CYP97A), cytochrome P450 family 97 B (CYP97B), and cytochrome P450 family 97 C (CYP97C) contribute to the production of lutein, a yellow pigment. During the synthesis of lutein, an intermediate compound called α-carotene is formed, resulting in an orange pigment. The other branch of the pathway involves lycopene, LCYB, β-carotene hydroxylase (BCH), zeaxanthin epoxidase (ZEP), and neoxanthin synthase (NSY) and leads to the formation of neoxanthin, another yellow pigment. Additionally, intermediate compounds such as antheraxanthin and violaxanthin can be transformed into red-colored capsanthin and capsorubin by capsanthin–capsorubin synthase (CCS). Only a few transcription factors have been identified to regulate carotenoid biosynthesis in flowers. For instance, coronatine insensitive 1, reduced carotenoid pigmentation 1, reduced carotenoid pigmentation 2, and white petal 1 transcription factors have been found to influence carotenoid biosynthesis in tobacco, monkeyflowers, and barrel medic (Wang et al. 2014; Sagawa et al. 2016; Meng et al. 2019).

The anthocyanin pathway is a well-understood and highly conserved pathway across different plant species (Zhang et al. 2014; Liu et al. 2021; Davies et al. 2022). The pathway can be divided into 3 main sections: the general phenylpropanoid pathway, the flavonoid pathway, and the anthocyanin-specific pathway (Jaakola et al. 2002; Zhang et al. 2014; Nguyen et al. 2023). The general phenylpropanoid pathway converts phenylalanine to cinnamate and then *p*-coumaroyl-CoA through a series of enzymatic reactions with phenylalanine ammonia lyase (PAL), cinnamate-4-hydroxylase (C4H), and 4-coumarate:CoA ligase (4CL; Liu et al. 2021). The molecule then enters the flavonoid pathway where it is transformed into dihydroflavonol with the help of chalcone synthase (CHS), chalcone isomerase (CHI), and flavanone 3-hydroxylase (F3H). Various flavanols are produced from each intermediate. The pathway diverges at dihydroflavonol, where either flavanone 3′,5′-hydroxylase (F3′5′H) or flavanone 3′-hydroxylase (F3′H) is expressed to direct the molecule toward the production of delphinidin-3-glucoside (purple/blue) or cyanidin-3-glucoside (red), respectively. Once a pathway is chosen, dihydroflavonol 4-reductase (DFR), anthocyanidin synthase (ANS), and UDP-glucose flavonoid 3-*O*-glucosyltransferase (UFGT) work together to produce the anthocyanin molecules. *O*-methyl transferases can also modify these anthocyanins to form other anthocyanin compounds such as peonidin glucoside.

Two studies have investigated the presence of anthocyanins in lantana flowers. In 1 study, researchers used liquid chromatography to extract anthocyanins and carotenoids from a lantana accession whose flowers transitioned from yellow to magenta after opening (Ram et al. 1984). They found that β-carotene was the major carotenoid present, while the major anthocyanin was delphinidin monoglucoside. The study collected flower samples at different stages of development and found that carotenoid synthesis was highest in unopened flowers, while anthocyanin biosynthesis was highest shortly after the flowers had opened.

In another study, the floral anthocyanins of 43 lantana plants with flowers ranging from orange to magenta were investigated using high-performance liquid chromatography (Huang et al. 2009). The researchers detected 6 anthocyanin pigments across the samples, including cyanidin 3-glucoside, 3,5-diglucoside, and 3-malonylglucoside, as well as peonidin 3,5-diglucoside and 3-malonylglucoside. Notably, cyanidin 3-glucoside and cyanidin 3-malonylglucoside were the most abundant pigments, present in all samples except for one. This 1 sample, which had white flowers, did not contain any anthocyanins. Interestingly, the authors did not find delphinidin glucoside to be a major anthocyanin in these samples, in contrast to the previous study.

Taken together, the 2 studies discussed above have provided valuable insights into the composition of anthocyanins and carotenoids in lantana flowers. However, to date, no research has been published on the genes responsible for the biosynthesis of these compounds in lantana. Understanding the structural and regulatory genes that have the greatest impact on pigment production can provide valuable information for breeders in their breeding programs. Therefore, the objective of this study is to investigate the genetic factors that influence the biosynthesis and accumulation of these pigments in various lantana cultivars with distinct flower colors and at different stages of floral development. By gaining a deeper understanding of the genetic factors that influence pigment production, we can facilitate the development of lantana cultivars with desired color traits and help to elucidate the underlying mechanisms that regulate anthocyanin and carotenoid biosynthesis in this species.

## Materials and methods

### Plant materials

Three diploid (2*n* = 2*x =* 22) *L. camara* varieties, “Lola,” “Denholm White,” and UF-T48, were propagated from cuttings and grown in 7.57-L pots filled with soilless potting mix for 5 months, from March to August. The plants were placed outdoors under 40% shade on metal benches arranged in a randomized complete block design with 3 replicates. Samples were collected at 2 stages: unopened flowers, which were 1–2 days prior to anthesis, and newly opened flowers, which were 0–1 days after anthesis. Before harvest, unopened, newly opened, and mature flowers (2–3 days post anthesis) were compared with the 1986 Royal Horticulture Society Color Chart to assign color codes representing the hue, saturation, and brightness of each flower. Individual flowers were then harvested from several inflorescences of each replicate, immediately frozen in liquid nitrogen, and stored at −80°C for subsequent analysis.

### RNA extraction and sequencing

Total RNA was extracted from the flower tissue of individual replicates using the Qiagen RNeasy Plus mini kit (Qiagen, Hilden, Germany) following the manufacturer's instructions after grinding in liquid nitrogen using a mortar and pestle. The RNA quality and quantity was assessed using the Nanodrop 8000 Spectrophotometer (Nanodrop Technologies, Wilmington, DE, USA), Qubit 4 Fluorometer (Waltham, MA, USA), and Agilent bioanalyzer 2100 (Agilent Technologies, Santa Clara, CA, USA). Sequencing library preparation and sequencing were performed by the University of Florida's Interdisciplinary Center for Biotechnology Research (ICBR, Gainesville, FL, USA). Equal parts of all 18 samples were pooled and submitted for two 8 M SMRT cells of PacBio IsoSeq sequencing on a Sequel II PacBio sequencer following the manufacturer's protocols (PacBio, Menlo Park, CA, USA). All 18 samples were also submitted individually for 8 GB of Illumina paired-end (150 bp) sequencing on a NovaSeq 6000 sequencer (Illumina, San Diego, CA, USA).

### De novo transcriptome assembly

PacBio IsoSeq reads were cleaned and assembled using the IsoSeq3 pipeline (PacBio 2020). Prior to clustering, reads from both PacBio flow cells were merged to generate highly polished isoforms with a predicted accuracy of ≥99%. For the raw Illumina reads, Trimmomatic 0.39 (Bolger et al. 2014) was utilized to perform trimming, followed by quality assessment with FastQC 0.11.7 (Andrews 2010). A de novo assembly of the Illumina reads was then conducted using Trinity 2.11.0 (Grabherr et al. 2011), which was subsequently merged with the PacBio transcriptome assembly. To create the reference transcriptome, redundant transcripts were removed using CD-HIT-EST 4.6.8 (Li and Godzik 2006) with an identity threshold of 0.95 and transcripts <200 bp were eliminated. Transcript completeness was assessed by using BUSCO 4.1.4 (Manni et al. 2021) against the eudicots_obd10 database of 2,326 genes.

### Transcriptome annotation

To predict CDS within the assembled reference transcriptome, Transdecoder 5.5.0 (https://github.com/TransDecoder/TransDecoder) was used. The resulting coding transcripts were annotated with predicted protein descriptions, gene ontologies (GOs), KEGG pathways, and other functional annotations using eggNOG-mapper 2.1.6 (Cantalapiedra et al. 2021). Four candidate genes identified in the study were used for phylogenetic map development to evaluate the quality of the automated annotations in MEGA 11 (Tamura et al. 2021). The tree was constructed using the neighbor-joining method with 1,000 replicates. WEGO 2.0 (Ye et al. 2018) was used to visualize the GO annotations in a graphical format, allowing for a more comprehensive characterization of gene functions. The online program g:Profiler version:e110_eg57_p18_4b54a898 (Kolberg et al. 2023) was used for GO enrichment and the identification of under- and over-represented gene clusters.

### Differential gene expression

Trimmed Illumina reads were mapped to the reference transcriptome using RSEM 1.1.3 (Li and Dewey 2011) to generate bam alignment files. These files were filtered and clustered into pseudo genes using Corset 1.0.6 (Davidson and Oshlack 2014) to reduce the impact of poorly mapped reads and obtain a more accurate read count matrix. The count matrix was loaded into edgeR 3.40.0 (Robinson et al. 2010) in R 4.2.2 (R Core Team 2022) to normalize the read counts and perform differential expression analysis. Differentially expressed genes (DEGs) were defined as those having a false discover rate <0.05 and an absolute value of logFC ≥ 2 (natural logarithm). To identify DEGs involved in the anthocyanin and carotenoid pathways, we used predicted protein names, GOterms, and KEGG pathways as annotations to extract relevant genes for comparative analysis.

### Quantitative real-time PCR analysis

To verify the expression levels of candidate genes identified by RNA sequencing, qRT-PCR was performed using the same RNA samples that were used for sequencing. First-strand cDNA was synthesized from 1 µg of total RNA using the High-Capacity RNA-to-cDNA Kit (Thermo Fisher, Waltham, MA, USA). Primers for each candidate gene and a reference gene (*EF1A*) were designed using Primer3 (Untergasser et al. 2012) and verified for specificity using standard PCR and 1% agarose gel electrophoresis. The qRT-PCR analysis was performed on an AriaMx Real-Time PCR system (Agilent Technologies) using PowerUP SYBR Green Master Mix (Thermo Fisher) according to the manufacturer's protocols. The protocol suggested by Daamgard and Treebak (2022) was followed for calculating adjusted 2^−ΔΔ*CT*^ values. Three technical replications were performed per sample.

## Results

### Flower color assessment

**Fig. 1.**
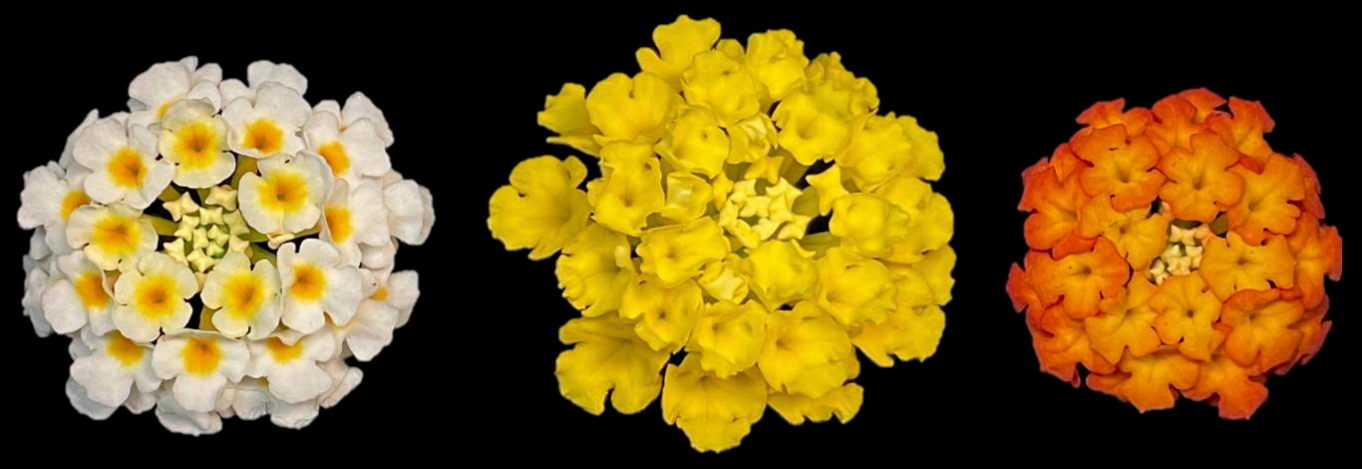
Representative flowers of Denholm White, Lola, and UF-T48 flowers (left to right) that were used for RNA extraction.

The flower color of the 3 lantana accessions was visually assessed as follows: Lola had yellow flowers, Denholm White had white flowers with an orange ring on the inside of the flowers, and UF-T48 had light orange flowers transitioning to red (Fig. 1). Using the Royal Horticultural Society Color Chart (RHSCC 1986), color codes ranging from 9A to 158D were assigned to each accession (Table 1). While some darkening of color was observed during flower aging in Lola, only UF-T48 showed a noticeable color transition from yellow to red throughout flower aging.

**Table 1.** Royal Horticultural Society Color Chart readings from 3 flower maturity stages in lantana.

Cultivar/line	Unopened flower color	Newly opened flower color (outer petal/inner petal)	Mature flower color (outer petal/inner petal)
Denholm White	Yellow-Orange 19C	Yellow-White 158D/Yellow-Orange 23A	Yellow-White 158D/Yellow-Orange 23A
Lola	Yellow 13A	Yellow 9A/Yellow 9A	Yellow 9C/Yellow 9C
UF-T48	Yellow-Orange 21B	Yellow-Orange 23A/Yellow-Orange 23B	Red 46A/Red 46A

Flower stages are unopened flowers (1–2 days prior to anthesis), newly opened flowers (0–1 days after anthesis), and mature flowers (2–3 days post anthesis).

### RNA sequencing and de novo assembly

The study performed full-length transcriptome sequencing using a PacBio Sequel II system, because of the lack of a reference genome for Lantana or closely related species. A total of 667,965 HiFi reads were generated, with a mean read length of 1,701 bp, for de novo flower transcriptome assembly (Supplementary Table 1). Furthermore, 517,603,414 Illumina paired-end short reads (150 bp) from 18 flower petal samples were obtained for de novo assembly and gene expression analysis (Supplementary Table 2). After trimming and filtering, the full-length IsoSeq reads were clustered into 13,583 consensus isoforms, comprising 23,415,989 bp with an N50 of 1,996 bp. Short Illumina reads were assembled into 1,552,655 transcripts with an N50 of 1,502 bp. The 2 assemblies were combined, and redundant transcripts were filtered out using CD-HIT to produce the reference transcriptome containing 718,058 transcripts with an N50 of 1,059 bp. The completeness of the reference transcriptome was assessed by using BUSCO, which showed 94.2% completeness. Among complete BUSCOs, 21.7% were single copy, while 72.5% were duplicated.

**Fig. 2.**
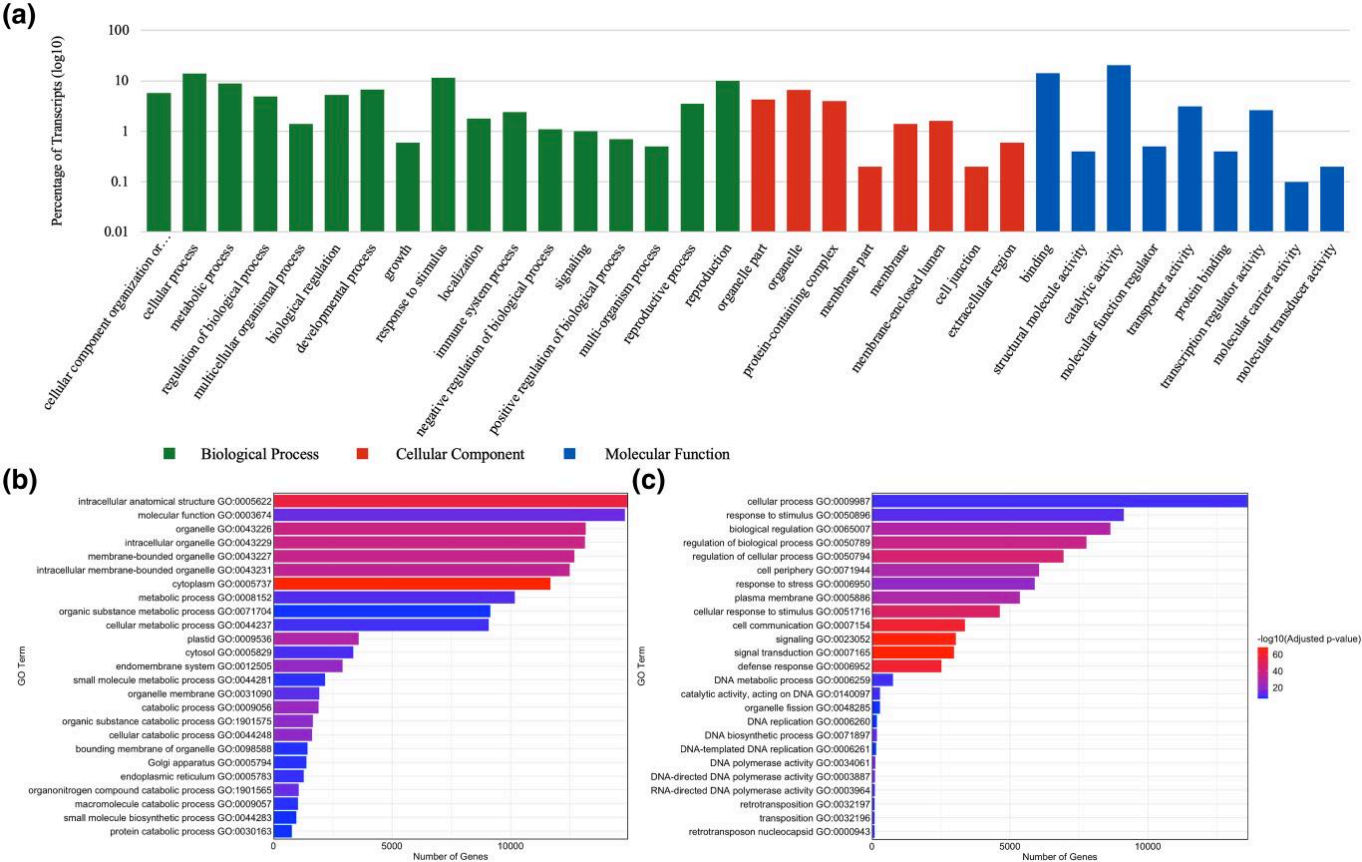
GO enrichment results of lantana flower transcriptome. a) GO enrichment histogram of all 23,336 gene clusters that were assigned GO terms in the lantana flower reference transcriptome. b) The top 25 significantly overrepresented GO terms among the DEGs in the lantana flower transcriptome. c) The top 25 significantly underrepresented GO terms among the DEGs in the lantana flower transcriptome.

From this transcriptome, a total of 313,998 coding sequences (CDSs) were identified using TransDecoder, with 154,905 being complete CDS, and 3,354 measuring ≥1 kb in length. The majority (91.13%) of CDSs ranged from 100 to 500 bp in length, and the largest CDS measured 5,067 bp in length. The CDSs were further annotated using eggNOG-mapper, resulting in the successful annotation of 279,533 CDSs. After filtering out CDS with nonplant protein hits and duplicate annotations, 124,100 annotated transcripts remained. This filtering step ensured that the remaining transcripts were plant-specific and would not interfere with downstream analyses. Nonplant protein hits were also removed from the reference transcriptome, bringing the total number of transcripts to 665,763. Alignment of reads to the transcriptome and clustering of transcripts using Corset produced 123,492 gene clusters, which grouped together transcripts that may be different isoforms or alternative splicing events of the same gene, providing a more comprehensive picture of the transcriptome. An analysis of gene GO annotations using WEGO revealed that the highest number of transcripts were involved in catalytic activity (Fig. 2a).

### Differential expression analysis

**Fig. 3.**
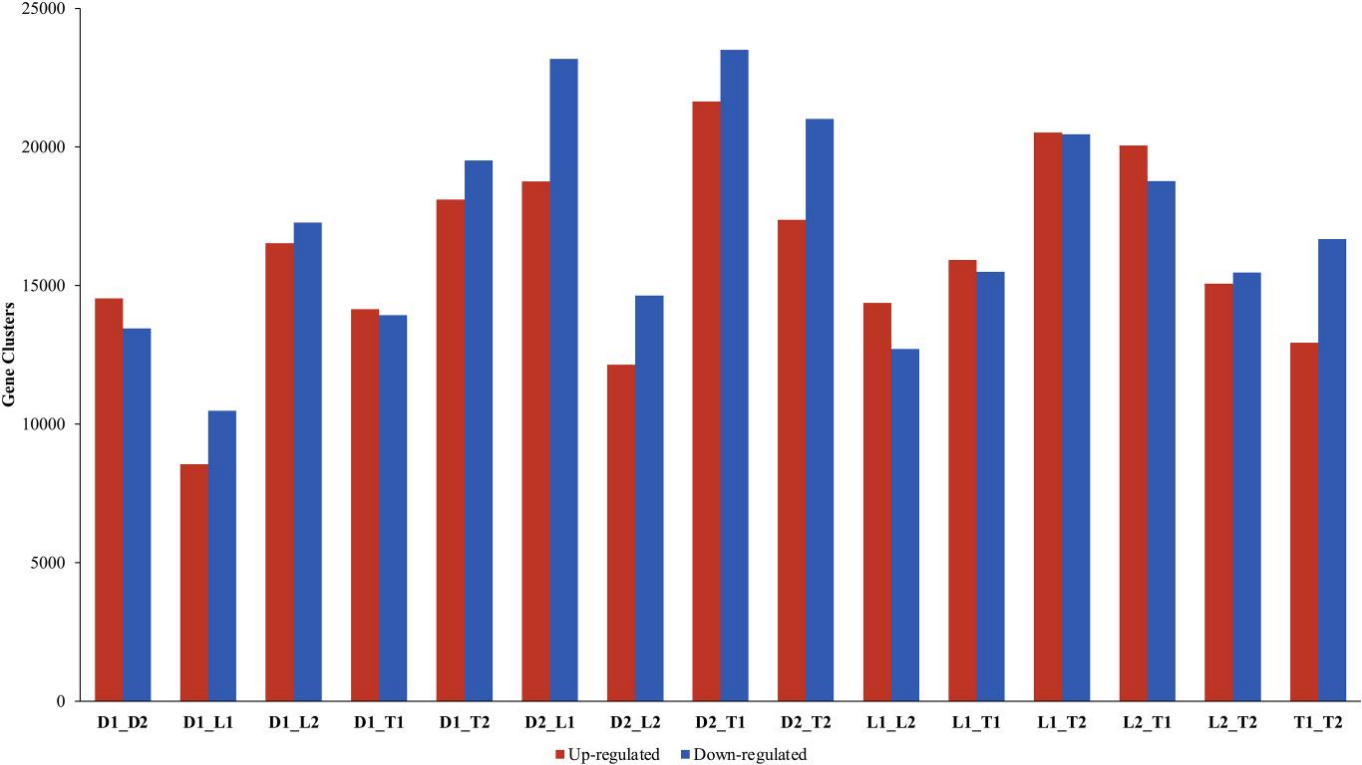
A bar chart representing the number of DEG clusters. Each set of bars represents all pairwise comparisons of unopened (1) and newly opened (2) lantana flowers of Denholm White (D), Lola (L), and UF-T48 (T) which exhibit white (with yellow inner petal), yellow, and red flowers, respectively.

The differential expression analysis was conducted on opened and unopened flowers from 3 lantana accessions, resulting in the identification of 72,862 unique gene clusters that were differentially expressed in 15 pairwise comparisons (among 3 flower colors and 2 developmental stages). On average, each combination of samples showed differential expression in 33,148 gene clusters (Fig. 3). The pairwise comparisons between opened and unopened flowers of the 3 lantana accessions revealed that the highest number of DEGs was observed between Denholm White opened flowers and UF-T48 unopened flowers, with 21,639 upregulated and 23,509 downregulated clusters. In contrast, the fewest DEGs were found between Denholm White unopened flowers and Lola unopened flowers, with only 8,554 upregulated and 10,476 downregulated clusters. The differential expression of 14,603 gene clusters could be successfully annotated to the protein level. A total of 23,336 gene clusters could be annotated with GO terms. A GO enrichment analysis of the DEGs revealed several overrepresented GO terms related to various biological processes. Among these, “cytoplasm” (GO:0005737) and “plastid” (GO:0009536) were notably overrepresented, as shown in Fig. 2b. The GO enrichment analysis also highlighted several GO terms that were significantly underrepresented. These included terms related to signal transduction, cellular communication, defense responses, DNA metabolism, and retrotransposition processes, among others (Fig. 2c).

### Analysis of anthocyanin biosynthesis genes

To identify gene clusters associated with anthocyanin biosynthesis, gene annotations were analyzed. Trimmed mean of *M* (TMM) values normalized read counts were produced before the DEG expression analysis. All samples were compared with UF-T48 opened and unopened flowers to analyze anthocyanin-related genes. Candidates of all 10 structural genes of the anthocyanin pathway were found in the assembled transcriptome (Supplementary Table 3). Among these, 8 core candidate anthocyanin biosynthesis genes were significantly upregulated in UF-T48 opened and unopened flowers, along with 2 highly upregulated transcription factors (Supplementary Table 4).

**Fig. 4.**
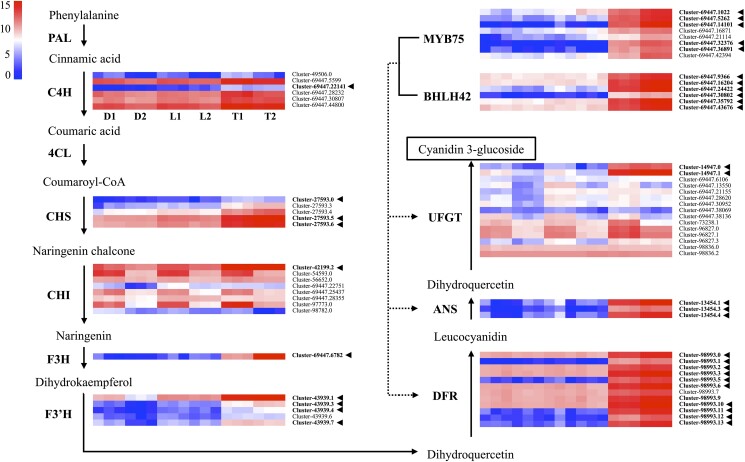
Anthocyanin biosynthesis pathway illustrated alongside heatmaps of log_2_-transformed TMM normalized counts for DEGs associated with enzymes in the pathway. These enzymes encompass PAL, 4CL, CHS, CHI, F3H, F3′H, DFR, ANS, and UFGT. Heatmap sample labels denote unopened (1) and newly opened (2) lantana flowers from Denholm White (D), Lola (L), and UF-T48 (T) genotypes, which exhibit white (with yellow inner petal), yellow, and red flowers, respectively. The color gradient in the heatmap, spanning red to blue via white, signifies upregulated, stably expressed, and downregulated genes. Gene clusters differentially expressed across all combinations featuring UF-T48 are emphasized in bold and indicated with an arrowhead. The dotted lines suggest potential genes activated by transcription factors.

Among the DEGs upregulated in all pairwise comparisons that included the UF-T48 samples, 1 gene cluster showing homology to *C4H* was consistently upregulated, while 4 other gene clusters were upregulated in certain sample combinations (Fig. 4). The white vs red flower comparisons showed almost double the log fold change (logFC) compared with yellow vs red flowers for this *CH4*-like gene cluster. All 5 gene clusters with homology to *CHS* identified were upregulated in all combinations at lower levels (<4 LogFC). Among the 8 gene clusters homologous to *CHI* found in the transcriptome, only 1 had elevated expression levels in UF-T48 samples, while several others had slight upregulation in white and yellow flowers. Only 1 gene cluster with homology to *F3H* was annotated from the transcriptome, and it was highly upregulated in red flowers, with logFC ranging from 4.6 to 9.2. Among the 6 annotated gene clusters with homology to *F3′H*, 5 were upregulated in red flowers, with each consistently showing a larger logFC in white vs red flowers. All 12 gene clusters homologous to *DFR* were upregulated in red flowers, with 5 of those clusters averaging >8.7 logFC in all combinations. The *ANS*-like gene clusters consisted of 3 clusters that were all highly upregulated, with an average of 9.58 logFC. Fifteen gene clusters with homology to *UFGT* were identified, but only 2 were differentially expressed in all comparisons, and 1 cluster was downregulated only in white-flowered samples and not in yellow ones.

In addition to the structural genes, 7 out of 8 gene clusters with homology to the transcription factor *MYB75* (*PAP1*) were upregulated in UF-T48, and 4 of those had a logFC >5. One of these *MYB75*-like clusters (Cluster-69447.14101) had the highest average logFC among all anthocyanin-related clusters with an average of 10. Another transcription factor, *bHLH42* (*TT8*), was present in 6 gene clusters that were all upregulated in UF-T48.

Although very few significant DEGs were identified between Denholm White and Lola samples, there was a general trend of slightly higher expression patterns of anthocyanin pathway genes in Lola flowers. Moreover, differences in the expression of anthocyanin structural genes between opened and unopened flowers were observed in Lola and UF-T48, with both varieties showing slight increases in expression (Fig. 4). However, the changes in expression were more pronounced in UF-T48.

Gene clusters homologous to *ANS*, *DFR*, *TT8*, and *PAP1* were aligned with corresponding gene homologs from model species to validate the automated annotations. These alignment data were then utilized to construct phylogenetic trees for each gene, as depicted in Supplementary Fig. 1.

### Carotenoid biosynthesis gene expression

**Fig. 5.**
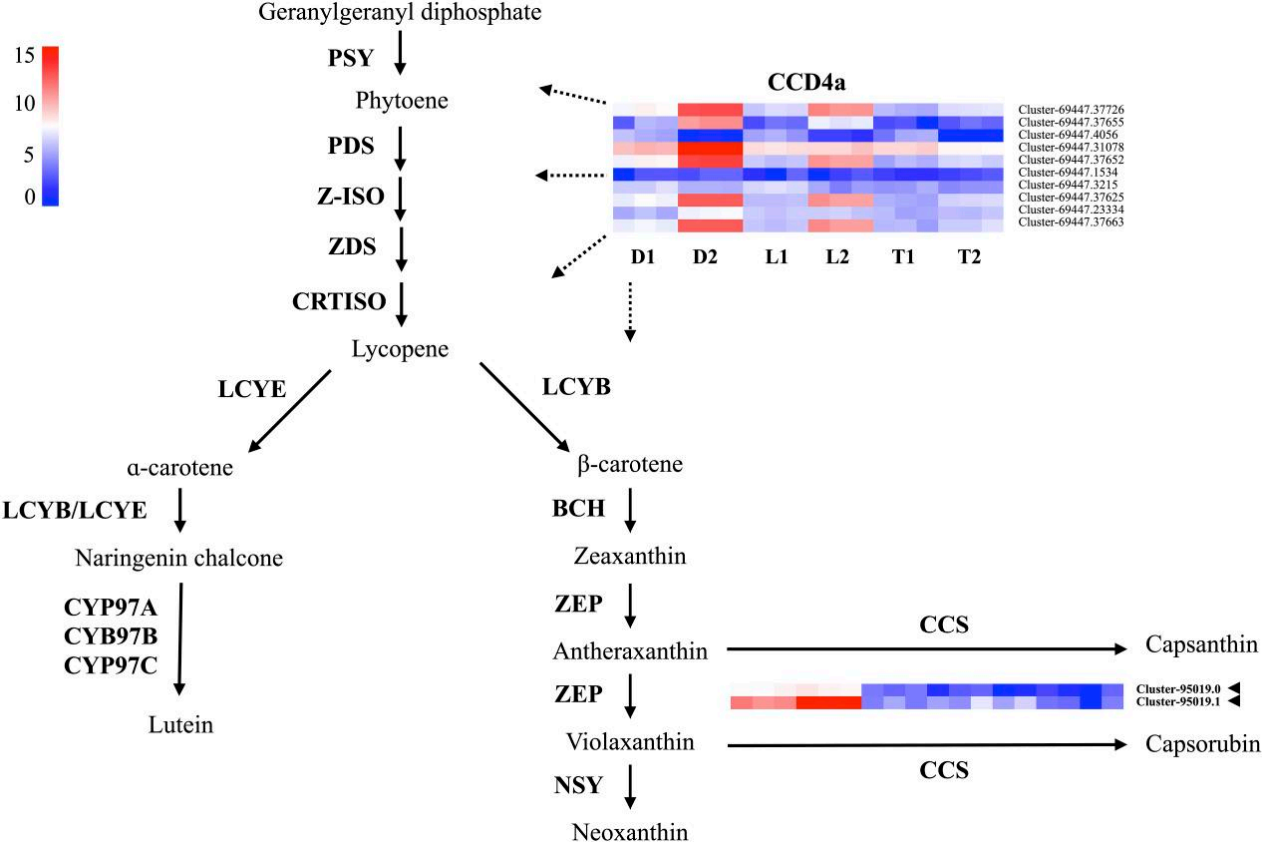
Carotenoid biosynthesis pathway illustrated alongside heatmaps of log2-transformed TMM normalized counts for DEGs associated with enzymes in the pathway. These enzymes encompass PSY, PDS, Z-ISO, ZDS, CRISTO, LCYE, LCYB, CYP97A, CYP97B, CYP97C, BCH, ZEP, NSY, and CCS. Heatmap sample labels denote unopened (1) and newly opened (2) lantana flowers from Denholm White (D), Lola (L), and UF-T48 (T) genotypes, which exhibit white (with yellow inner petal), yellow, and red flowers, respectively. The color gradient in the heatmap, spanning red to blue via white, signifies upregulated, stably expressed, and downregulated genes. Gene clusters differentially expressed across all combinations featuring Denholm White are emphasized in bold and indicated with an arrowhead. The dotted lines suggest that the specific part of the pathway regulated by the transcription factor remains unidentified.

To investigate the potential role of carotenoids in lantana flowers, gene clusters associated with carotenoid biosynthesis were extracted using gene annotations. A comparison was made between the gene expression levels in the Lola (yellow) and UF-T48 (orange to red) samples with those in the Denholm White (white) sample to identify DEGs. Although the reference transcriptome contained annotations for many core carotenoid biosynthesis genes (Supplementary Table 5), few were found to be differentially expressed. However, a specific gene cluster, annotated as a carotenoid cleavage dioxygenase (*CCD4a*), was found to be highly upregulated in opened white flowers, and to a lesser extent, yellow flowers (Fig. 5, Supplementary Table 6). Furthermore, 2 candidate *CCS* gene clusters were also highly upregulated in white flowers and were differentially expressed in all 8 pairwise combinations of samples with Denholm White (3 flower colors and 2 developmental stages).

### Validation of RNA-seq using qRT-PCR

**Fig. 6.**
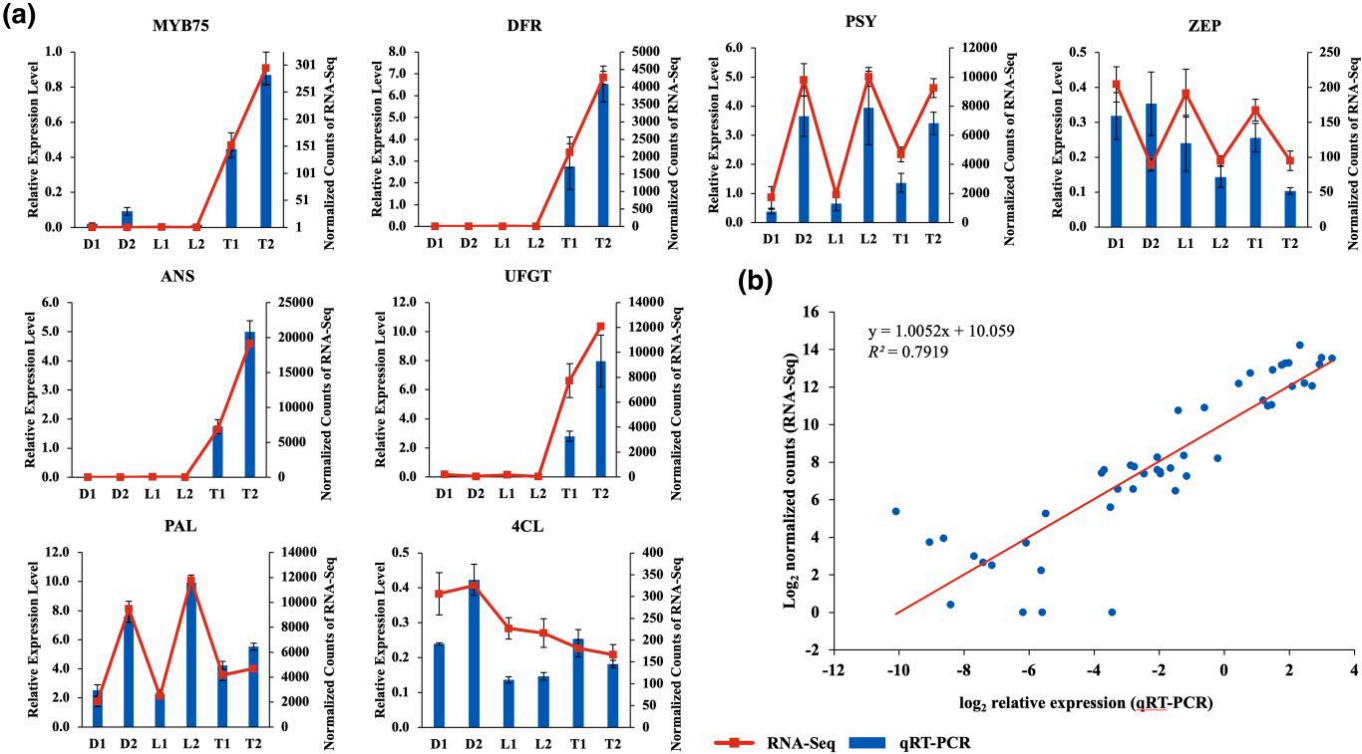
qRT-PCR validation of gene expression in the lantana flower transcriptome. a) qRT-PCR and RNA-seq expression levels in the transcriptome. The *x*-axis labels denote unopened (1) and newly opened (2) lantana flowers from Denholm White (D), Lola (L), and UF-T48 (T) genotypes, which exhibit white (with yellow inner petal), yellow, and red flowers, respectively. Eight genes were selected for qRT-PCR: *DFR*, *ANS*, *UFGT*, *MYB75*, *PAL*, *4CL*, *PSY*, and *ZEP*. b) Correlation analysis of the expression values between qRT-PCR and RNA-seq.

To validate expression profiles generated by RNA-seq data, 4 highly upregulated gene clusters with homology to *DFR* (Cluster-98993.11), *ANS* (Cluster-13454.4), *UFGT* (Cluster-14947.1), and *MYB75* (Cluster-69447.14101) were selected for qRT-PCR assays (Supplementary Tables 7 and 8). In addition, 4 gene clusters that were stably expressed across all samples with homology to anthocyanin and carotenoid genes *PAL*, *4CL*, *PSY*, and *ZEP* were included in the qRT-PCR assay. The expression levels were normalized to the expression of *EF1A* and compared with the RNA-seq data (Fig. 6a). Linear regression analysis suggests that the expression levels for these genes were correlated with the RNA-seq results (*R*^2^ = 0.79), indicating that the anthocyanin candidate genes were indeed upregulated in UF-T48 (Fig. 6b).

## Discussion

Lantana is a popular ornamental plant due to its vibrant and diverse flower colors, but the genetic basis of these colors is not well understood. Despite the importance of this plant, only a limited number of genetic studies have been carried out (Peng et al. 2019; Shah et al. 2020). In this study, a comprehensive analysis of the transcriptome of 3 lantana accessions using full-length transcriptome sequencing was performed, which allowed the identification of DEGs associated with flower color. The de novo assembly of the transcriptome yielded a rich resource of genes involved in various biological processes, with a particular focus on genes associated with anthocyanin and carotenoid biosynthesis. This study also identified 2 highly upregulated transcription factors that potentially play key roles in the regulation of anthocyanin biosynthesis. These findings provide valuable insights into the genetic basis of flower color development in lantana and can be used to develop new cultivars with improved flower colors. Furthermore, this study provides a resource for future genetic and functional studies of lantana and related species.

In this study, the de novo assembly produced a notably high transcript count of 665,763. Several factors might underlie this elevated number. Mis-assemblies, particularly at the 3′ and 5′ ends of transcripts, can artificially augment the count of unique sequences. Moreover, alternative splicing, a common phenomenon in eukaryotes, can generate multiple transcript variants from a single gene, further amplifying the count. Another potential contributor is RNA contamination from other organisms such as insects or pathogens. While nonplant transcripts that could be annotated were removed, those unidentifiable by the automatic annotation software persisted in the dataset. It is noteworthy that the postassembly processing, especially the clustering step, considerably reduced the transcript count. This suggests that many initial sequences might be variants or fragments of the same gene. However, not all splice variants clustered effectively, possibly because of noncoding sequences at the 5′ and 3′ transcript ends. This is evident in candidate genes like TT8, where multiple clusters exist, but only 2 gene forms are apparent in the nucleotide sequence alignments (Supplementary Fig. 2). The presence of a high-quality reference genome in the future would allow for easier, reliable classifications of these transcripts.

Flower color development is well understood in some other ornamental crops, such as chrysanthemums (Noda et al. 2013), magic flowers (Roberts and Roalson 2017), and peonies (Zou et al. 2023), but in lantana, little is known about the genes responsible for producing the various vibrant colors in the species. Among the top 25 overrepresented GO terms in the DEGs in this study were cytoplasm (GO:0005737) and plastid (GO:0009536), in which anthocyanins and carotenoids are, respectively, produced (Zhao et al. 2022). The core anthocyanin biosynthesis genes play crucial roles in the synthesis of pigments responsible for various colorations in plant tissues, including flowers, fruits, and leaves. In many plants, the key upregulated genes typically include *CHS*, *CHI*, *F3H*, *F3′H*, *DFR*, *ANS*, and *UFGT* (Tanaka et al. 2008; Petroni and Tonelli 2011; Nguyen et al. 2023). For example, in petunia (*Petunia hybrida*), the upregulation of *CHS*, *DFR*, and *ANS* was found to be crucial for the accumulation of anthocyanins in corolla lobes (Quattrocchio et al. 1999). In *Camellia sinensis*, expression analysis in flowers revealed that *PAL*, *4CL*, *F3H*, *DFR*, and *UFGT* genes were highly expressed, contributing to the accumulation of anthocyanins (Zhou et al. 2020; Maritim et al. 2021). *DFR* was found to be the most highly upregulated anthocyanin pathway gene in red *Lycoris radiata* flowers (Wang et al. 2021). The results of this study show a clear association between the expression of anthocyanin biosynthesis genes and the color variation observed in the lantana flowers. The red-flowered UF-T48 showed an upregulation of several gene clusters with homology to key anthocyanin pathway genes, such as *CHS*, *CHI*, *F3H*, *F3′H*, *DFR*, *ANS*, and *UFGT*, compared with the white and yellow-flowered accessions. However, consistent with *L. radiata*, a *DFR* homolog was the highest expressed among the core anthocyanin pathway genes.

Additionally, gene clusters with homology to the transcription factors *MYB75* and *bHLH42*, which have been previously reported to regulate anthocyanin biosynthesis in other plant species (Gonzalez et al. 2008; Xu et al. 2015), are also upregulated in UF-T48. In *Arabidopsis thaliana*, the overexpression of the *PAP1* (*MYB75*) transcription factor resulted in the upregulation of *CHS*, *CHI*, *F3H*, *F3′H*, *DFR*, *ANS*, and *UFGT* genes, leading to higher anthocyanin accumulation (Borevitz et al. 2000). *TT8* (*bHLH42*) has also been shown to interact with *PAP1* to activate core anthocyanin genes in *Freesia hybrida*, Arabidopsis, and tobacco (Li et al. 2020). These findings, in conjunction with previous research, are consistent with the idea that the red coloration in UF-T48 flowers might be influenced by the activation of core anthocyanin biosynthesis genes, including *DFR*, potentially regulated by *PAP1* and *TT8*. However, direct functional validation in UF-T48 is needed to confirm this hypothesis.

In contrast, only slight differences in the expression of anthocyanin pathway–related genes are observed between the Denholm White (white) and the Lola (yellow) accessions. This suggests that other factors such as post-translational modifications or the presence of other pigments might contribute to the color differences between these accessions (Tanaka et al. 2008; Davies et al. 2012). Moreover, the analysis revealed that the expression of candidate anthocyanin structural genes increases with flower aging in both Lola and UF-T48, which is in line with the observed color darkening in Lola and color transitioning in UF-T48 during flower development.

The investigation into carotenoid biosynthesis gene expression revealed few DEGs among the lantana accessions. However, a gene cluster with homology to the *CCD4a* gene is highly upregulated in opened white flowers and to a lesser extent in yellow flowers, suggesting a possible role in the development of white and yellow flower coloration (Ohmiya et al. 2006). In chrysanthemums, *CCD4a* was found to be highly expressed in white flower petals, suggesting its role in white flower color (Zhang et al. 2022). The overexpression of a *CCD4* gene in yellow petunia resulted in pale yellow flowers with reduced carotenoid content (Phadungsawat et al. 2020). Furthermore, 2 *CCS* candidate gene clusters are highly upregulated in white flowers, indicating a potential contribution to the unique orange color pattern observed on the inside of Denholm White flower petals. *CCS* genes have been shown to contribute to yellow and orange hues in flowers of other ornamental crops (Yang et al. 2014; Trajkovi et al. 2021).

### Conclusion

This study offers insights into the potential molecular mechanisms that might underlie flower color variation in lantana accessions. Through the de novo assembled transcriptome, we identified gene clusters that show homology to key genes in anthocyanin and carotenoid biosynthesis pathways, as well as potential associated transcription factors. The differential expression patterns observed among these gene clusters are consistent with known flower color phenotypes in the 3 lantana accessions, suggesting a possible correlation. While these findings provide a foundation for understanding the genetic underpinnings of flower coloration, direct functional validation is essential for making definitive conclusions. The insights from this study hint at potential applications in plant breeding programs and the ornamental plant industry, suggesting that, with further research, it might be possible to develop new lantana varieties with specific color patterns. The transcriptome and gene expression data generated here can also serve as a starting point for future research on lantana and related species. This study underscores the potential of RNA sequencing and de novo assembly in investigating complex traits in nonmodel organisms, especially in the absence of a reference genome. Future research can build upon these findings and explore other facets of lantana biology, such as pollination, growth, and development, using the transcriptomic resources provided in this work.

## Supplementary Material

jkad259_Supplementary_Data

## Data Availability

RNA-seq raw data and assembled transcriptome are available from the NCBI Sequence Read Archive BioProject database with the accession number PRJNA956917. DEG results and gene counts are available on figshare: https://doi.org/10.25387/g3.24318871. Supplemental material available at G3 online.
