# Supplementary material for: Transcriptome analysis of Lantana camara flower petals reveals candidate anthocyanin biosynthesis genes mediating red flower color development
Source: G3 (Bethesda). 2023 Nov 17;14(1):jkad259. doi: 10.1093/g3journal/jkad259 (PMC10755171; doi:10.1093/g3journal/jkad259)
Supplement: jkad259_Supplementary_Data [file jkad259_supplementary_data.zip › Supplementary_Material_Legends_G3-2023-404633[61].docx]

Supplementary material

**Supplementary figure S1:** Phylogenetic analysis of lantana candidate genes in relation to homologous genes from reference species involved in the anthocyanin biosynthetic pathway. The trees were constructed using the neighbor-joining method by the MEGA 11 software. The numbers indicate bootstrap values for 1,000 replicates. The GenBank accession numbers for each reference are in parenthesis after each species name. (A) Phylogenetic tree comparing candidate genes with PAP1 homologs from various species. (B) Phylogenetic relationship between candidate genes and ANS homologs from selected species. (C) Phylogenetic analysis showcasing the evolutionary relationship of candidate genes with TT8 homologs. (D) Phylogenetic tree illustrating the similarity between candidate genes and DFR homologs from different species.

**Supplementary figure S2:** Multiple sequence alignment of nucleotide sequences from differentially expressed gene clusters in the lantana flower transcriptome showing homology to the TT8 transcription factor. The depicted alignment corresponds to positions 1593-1683 of the gene cluster Cluster-69447.16204.

**Supplementary file S1: Supplementary table S1.** Sequencing statistics from PacBio Isoseq sequencing of lantana flower RNA samples. **Supplementary table S2.** Trimmomatic output of all 18 lantana flower RNA-Seq samples. Samples are denoted unopened (1) and newly opened (2) lantana flowers from ‘Denholm White’ (D), ‘Lola’ (L), and UF-T48 (T) genotypes, which exhibit white (with yellow inner petal), yellow, and red flowers, respectively. A, B, and C represent each biological replicate. **Supplementary table S3.** List of gene clusters and corresponding gene annotations for anthocyanin biosynthesis structural genes. **Supplementary table S4.** List of gene clusters that were differentially expressed in all red flower samples with corresponding LogFC (natural logarithm) for each pairwise comparison. Each column represents all pairwise comparisons of unopened (1) and newly opened (2) lantana flowers of ‘Denholm White’ (D), ‘Lola’ (L), and UF-T48 (T) which exhibit white (with yellow inner petal), yellow, and red flowers, respectively. **Supplementary table S5.** List of gene clusters and corresponding gene annotations for carotenoid biosynthesis structural genes. **Supplementary table S6.** List of gene clusters that were differentially expressed in all white and yellow flowered lantana samples with corresponding LogFC (natural logarithm) for each pairwise comparison. Each column represents all pairwise comparisons of unopened (1) and newly opened (2) lantana flowers of ‘Denholm White’ (D), ‘Lola’ (L), and UF-T48 (T) which exhibit white (with yellow inner petal), yellow, and red flowers, respectively. **Supplementary table S7.** List of primers used for qRT-PCR analysis. **Supplementary table S8.** Gene sequences of differentially expressed candidate genes in anthocyanin and carotenoid pathways within the lantana flower transcriptome.

**Supplementary file S2: Supplementary table S9.** Raw counts for each gene cluster for all 18 lantana flower samples. **Supplementary table S10.** TMM normalized counts for each gene cluster for all 18 lantana flower samples.

**Supplementary file S3: Supplementary table S11.** Differential expression results from each pairwise comparison between all 18 samples.

*Supplementary Figures S1, S2 and File S1: <https://doi.org/10.1093/g3journal/jkad259>

*Supplementary Files S2 and S3: <https://doi.org/10.25387/g3.24318871>
