# Supplementary figures and images for: Transcriptome analysis of Lantana camara flower petals reveals candidate anthocyanin biosynthesis genes mediating red flower color development
Source: G3 (Bethesda). 2023 Nov 17;14(1):jkad259. doi: 10.1093/g3journal/jkad259 (PMC10755171; doi:10.1093/g3journal/jkad259)

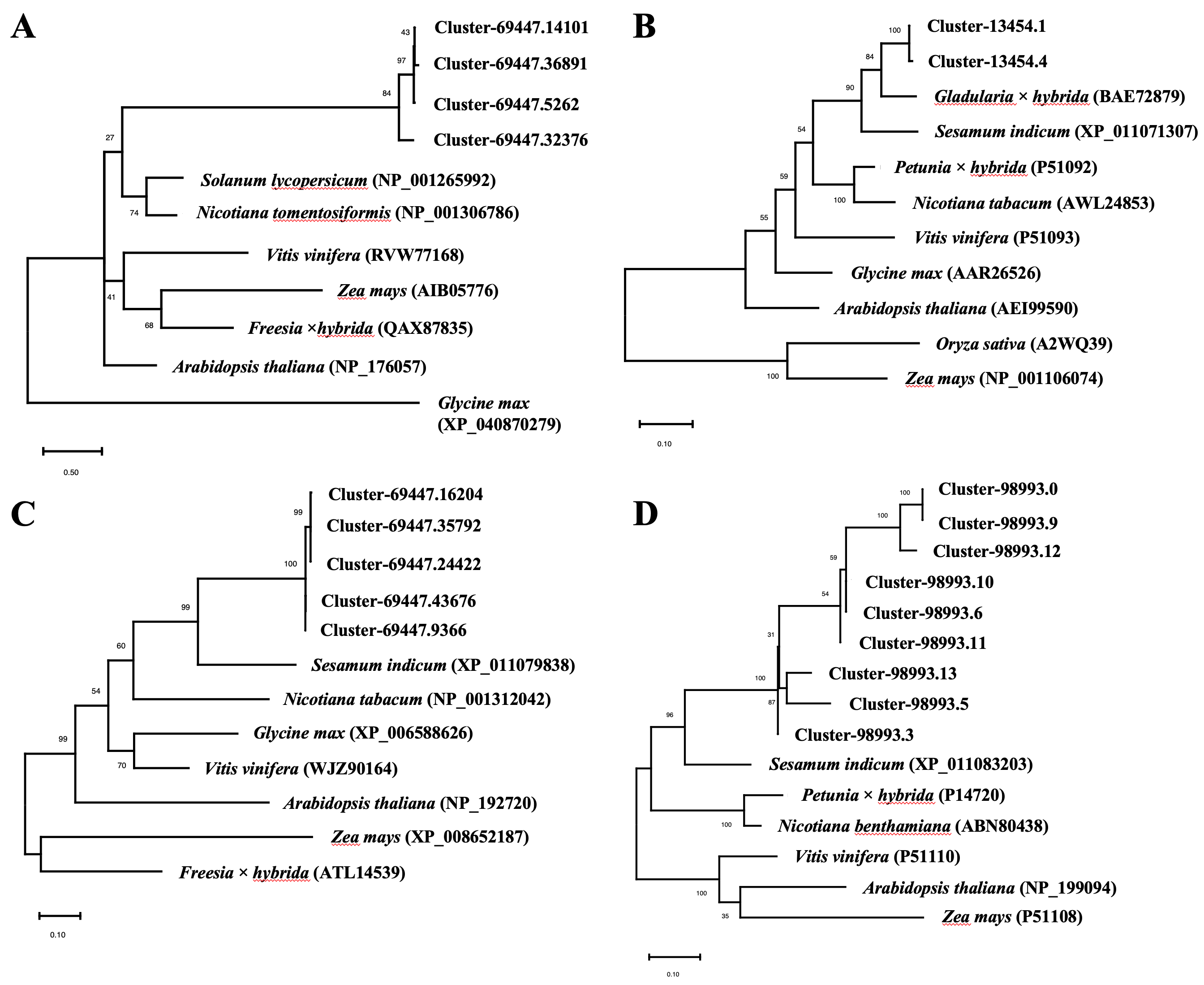

Supplement: jkad259_Supplementary_Data [file jkad259_supplementary_data.zip › Supplementary_Figure_S1_G3-2023-404633.png]

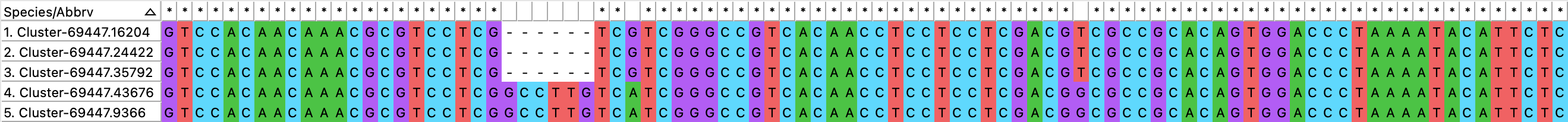

Supplement: jkad259_Supplementary_Data [file jkad259_supplementary_data.zip › Supplementary_Figure_S2_G3-2023-404633.png]
